# Capturing Optimal Mobile 2D Facial Images in Remote Aesthetics Medicine Clinical Trials: Technical Considerations for Facial Severity Analysis

**DOI:** 10.2196/64764

**Published:** 2026-01-28

**Authors:** Damon Caiazza, Scott Kreutzkamp

**Affiliations:** 1 AbbVie, 2525 Dupont Drive, Irvine, CA, 92612, United States, 1 614-946-7245

**Keywords:** forehead lines, lateral canthal lines, 2D facial image capture, consultation, remote, augmented reality guidance

## Abstract

**Background:**

In aesthetic clinical trials, image self-capture using mobile devices may help reduce burden on clinic resources, increase data quality, and lower barriers to study participation.

**Objective:**

This study aimed to develop a mobile device app to help participants self-capture clinically usable images.

**Methods:**

The Allergan Aesthetic (an AbbVie Company) mobile image app was designed to auto-capture images while directing study participants on distance, head position, and expression to capture a high-quality clinical image. To assess resolution and optimal lighting conditions, images captured using the app in office, at home, and in outdoor settings were compared with those from a Canfield VISIA-CR system (Canfield Scientific). Objective image quality assessment of facial images captured using the app with an iPhone XR (Apple Inc) and iPhone 12 (Apple Inc), compared with images captured using the Canfield VISIA-CR with a digital single-lens reflex camera and the Canfield mobile image capture app with a variety of Android (Google) and iOS (Apple Inc) devices, was conducted using the Blind/Referenceless Image Spatial Quality Evaluator (BRISQUE). Clinical utility was assessed by calculating inter- and intrarater variability for severity ratings of participants’ lateral canthal lines (LCL) or forehead lines (FHL) obtained from app-captured images compared with ratings based on in-person evaluations performed by a physician. Usability was assessed according to the ISO (International Organization for Standardization)/IEC (International Electrotechnical Commission) 250101 standard.

**Results:**

The Allergan Aesthetic mobile image app was found to perform best under natural light and had image resolution insufficient for assessing minor facial structures, but appropriate for larger structures (eg, FHL). A total of 3968 images were assessed using BRISQUE. Images captured with the Allergan Aesthetic mobile image app had better image quality than those captured using other modalities, as indicated by lower mean BRISQUE scores of 14.05‐19.81 compared with Canfield VISIA-CR with a DSLR (34.47) and the Canfield mobile image capture app (23.43). LCL and FHL were rated both in person and digitally in 68 and 71 participants, respectively (median age 52‐56 y; 48% to 52% female; 75% to 78% White). Interrater reliability between clinician live evaluations and independent photo review of self-captured photos based on intraclass correlation coefficients (ICCs) was substantial (0.61‐0.80) to almost perfect (0.81‐1.00) for all raters (LCL: ICC 0.75‐0.91 at rest and 0.79‐0.89 at maximum contraction; FHL: ICC 0.77‐0.93 at rest and 0.70‐0.89 at maximum contraction). After 2 iterations of improvements, mean usability ratings of the app experience (out of 5) were as follows: easy to complete=3.2, enjoyable=3.1, satisfied with the level of guidance provided=3.2, and likely to complete a full session without exiting=4.1.

**Conclusions:**

The Allergan Aesthetic mobile image app delivers consistent, high-quality images that allow for assessment of LCL and FHL in good agreement with in-person evaluation. Image self-capture using mobile devices may help reduce clinic costs and remove barriers to participation in aesthetic clinical trials.

## Introduction

The first documented use of photography in reconstructive surgery was in the late 1840s, and in recent decades, it has become a mainstay of aesthetic medicine [1,2]. Photography has been used for procedure planning, postprocedure assessment, and follow-up, as well as education [3,4]. In some settings, 3D imaging may be preferred over traditional 2D photography, but it remains expensive and often requires a large amount of office space [5].

The consistency of images can be affected by lighting conditions, such as use of ambient light, flash photography, or more complex setups, regardless of the exact mode of image capture [6,7]. For example, when using natural light, differences in lighting direction and intensity can make images taken at different times difficult to compare [8,9]. Furthermore, facial wrinkles can be washed out by light that is too strong [8]. These issues can have a significant impact on image assessment. Before the advent of digital photography, shutter speed, aperture, and film speed, as well as darkroom procedures, all had the potential to impact image consistency [2,3]. Although shutter speed and aperture remain important parameters in digital photography, further complexity and inconsistencies may be introduced through variations in sensor resolution and sensitivity, white balance, and processing of the image after capture [2,5].

There has been much effort in recent decades to maximize the consistency of image capture [2,3,8]. This includes adoption of specialized equipment, such as digital single-lens reflex (DSLR) cameras, 3D photography systems, and standardization of camera settings, lenses, lighting, and background [2-5,9,10]. Some clinical practices go as far as to set aside space for dedicated in-office studios, and even hire professional photographers [7,8,11]. All of these approaches require significant resource use in terms of equipment costs, space, and staff time, as well as the need for repeated office visits by patients.

The trend toward telemedicine and the desire to minimize office visits, driven by the COVID-19 pandemic, further increases the potential utility of mobile phone image capture in aesthetic medicine [12-14]. Almost everyone today has a good-quality camera integrated into their mobile phones. The utility of mobile phone cameras in medicine has already been shown to be effective in the teledermatology setting for assessment of clinically important skin conditions, such as skin cancer and psoriasis [15-17]. Despite this, some authors have cautioned against the use of mobile phone cameras due to their limitations [5]. One potential limitation of mobile phones is that, in most cases, the camera, if used as a camera app only, will automatically adjust settings to optimize the image [5]. However, recent advances in mobile phone operating systems provide increased controls over automatic adjustments, enabling the app to control image quality and consistency.

The objective of this study was to develop a mobile phone app with optimized and standardized camera settings that produce reproducible and consistent images that have clinical utility for medical professionals. Mobile imaging will allow patients to take photographs themselves in mobile-based aesthetic clinical trials, as well as drive efficiencies of cost and scale by reducing office time for both patients and clinic staff.

## Methods

### Mobile Image Capture App Development

A base set of criteria, including lighting, position (rotation, tilt, and distance), and expression, was established as the first steps in the development of the Allergan Aesthetic mobile image app (version 1.0; an AbbVie Company). Images were captured under a range of lighting conditions, including overhead lighting similar to an office setting (color range of 6000 Kelvin), incandescent lighting similar to an at-home setting (5000 Kelvin), and natural outdoor light on sunny days. These images were compared with those captured using a Canfield VISIA-CR system (Canfield Scientific). The optimal distance from the face was assessed over a range from 1 to 10 inches. Values for the position parameters were set with only a small degree of freedom, such that a greater than 5° difference prompted the user to reposition their head rotation or tilt. To achieve this, AR Foundations face tracking and recognition technology (Unity Technologies) was implemented; this approach allowed for images to be reproducibly captured from different aspects. Facial recognition technology was also used to help control for expression. In order to control for intersubject variability, as well as for the effects of treatments such as onabotulinumtoxinA and hyaluronic acid fillers, facial recognition was used to establish a baseline at the start of each session. This calibration tool also provides a facial outline in the app that acts as a guide to establish where the face should be (rotation and tilt) for each image. Expression staging was separated from other staging functions to limit screen prompts, thus preventing cognitive overload. Expression was assessed at the end of staging immediately before image capture. If the participants made the correct facial expression (eg, smile), the app asked the user to remain still and counted down to the final capture.

Images for all studies described here were auto-captured on an iPhone XR (Apple Inc) and an iPhone 12 (Apple Inc) using the mobile devices’ built-in software. Auto-capture was critical to ensure images were only collected (or captured) when staged within the tight parameters discussed above. Auto-capture also obviates the need for the participants to press a button, which can be difficult, especially when the desired image is not straight on. A built-in audio guide instructs the participants to adjust their position so they do not need to look at the screen. Key specifications for cameras used in these studies are listed in Table 1.

**Table 1.** Specifications of cameras used for image capture in this study.

Specification	Canfield VISIA-CR and Canon EOS 6D	iPhone XR	iPhone 12
Resolution, megapixels	20.2	7	12
Sensor size	35.8×23.9 mm	1/2.55 inch	1/3.6 inch
Pixel dimensions	3648×5472	1908 ×3392	2268×4032
Pixel size, μm	6.54	1.4	NR^a^
Aperture	f/16	f/2.2	f/2.2
Aspect ratio	3:2	16:9	16:9
Focal length, mm	24‐105	30	23

a NR: not reported.

### Image Quality Assessment

Objective image quality assessments were conducted using the Blind/Referenceless Image Spatial Quality Evaluator (BRISQUE; University of Texas) [18] within Matlab (Mathworks, Inc). In total, 4 sets of images were collected in a clinical setting and subjected to the BRISQUE algorithm to calculate a mean score, as well as maximum and minimum scores. The team collected images using 4 different sets of devices and software. The BRISQUE algorithm was then applied to images of faces under the different lighting conditions tested to calculate mean (minimum, maximum) scores. Lower scores indicated better image quality. Image set 1 was collected using the Allergan Aesthetic mobile image app (version 1.0) with an iPhone XR running iOS (iOS 14.2); image set 2 was collected using the Allergan Aesthetic mobile image app with an iPhone 12 running iOS (iOS 14.2.1); image set 3 was collected using the Canfield VISIA-CR Blue (version 7.5.4) with a DSLR camera (Canon EOS Rebel T3i; Canon); and image set 4 was collected using the Canfield mobile image capture application with a variety of Android and iOS devices.

### Clinical Analysis

Outputs from the Allergan Aesthetic mobile image app were assessed by a plastic surgeon and a clinical dermatologist (clinical photo raters) at a single site in the United States. This was a 3-part, noninterventional study. In order to ensure a wide range of facial types, there were no restrictions on entry criteria. Participants were recruited by a third party (Clinical Trial Media) from existing databases, online, and through traditional advertisements, such as flyers. In the first part of the study, physicians conducted a live in-person assessment of the severity of participants’ lateral canthal lines (LCL) and forehead lines (FHL). In the second part of the study, participants were randomized to have photographs taken using standard DSLR image capture or to self-capture images via the Allergan Aesthetic mobile image app. In the third phase of the study, the captured images were subjected to independent photo review by 2 clinical photo raters, a plastic surgeon and a clinical dermatologist, not involved in any other aspect of the study. Traditional photonumeric scales were used for the live rating and the standard photography for LCL and FHL; these included the Allergan Aesthetic scales at rest and maximum expression for FHL (forehead lines severity scale [FHLSS]) and LCL (lateral canthal lines severity scale [LCLSS]). The clinical photo raters were trained on the 4 photonumeric scales used in the study and asked to rate the severity of LCL and FHL at rest and at maximum contraction (ie, natural smile for the LCL and maximum brow elevation for the FHL). For measurement of intrarater reliability, rating of images was performed in 2 reviews (independent photo reviews 1 and 2) conducted at least 2 hours apart. Each clinician photo rater logged onto a secure web-based photo evaluation and data collection system to complete ratings of the photos in a prespecified random order. The results of independent photo review were then compared to in-person assessment in order to evaluate whether images from standard photography and self-capture using the mobile app are comparable to in-person assessment. Overall interrater reliability and intrarater reliability for the LCLSS and FHLSS among the clinician image raters were evaluated using the same statistical methods as those for clinician live raters. The one-to-one interrater reliability for the LCLSS and FHLSS between each clinician live rater and clinician image rater was evaluated using intraclass correlation coefficients (ICC) (2,1) at image validation 1 and separately at image validation 2. SAS macro “INTRACC” was used to calculate ICC (2,1). CIs were obtained based on the formula described by Shrout and Fleiss [19]. The estimated degree of agreement between raters was classified based on ICCs as follows: <0=poor, 0‐0.20=slight, 0.21‐0.40=fair, 0.41‐0.60=moderate, 0.61‐0.80=substantial, and 0.81‐1.00=almost perfect [20].

### Usability

Usability of the Allergan Aesthetic mobile image app (version 1.1) was assessed according to the ISO (International Organization for Standardization)/IEC (International Electrotechnical Commission) 250101 standard, which ensures “the degree to which a product or system can be used by specified users to meet their needs to achieve specific goals with effectiveness, efficiency, freedom from risk, and satisfaction in a specified context of use” [21]. Usability research was conducted by McDougald Research (Columbus, Ohio). After an initial round of research, feedback was addressed and iterative changes were made to the app; usability was then reassessed in a second round of research.

The study was divided into 2 main sections. The first was a priming activity, in which participants watched a video summarizing the research background and then were asked to download the app and attempt 2 capture sessions over the course of 3‐4 days. After completing both capture sessions, which were recorded using the Lookback.io platform (Lookback), participants rated the app in response to a series of questions (Multimedia Appendix 1). Participants rated the app on a 6-point scale from 0 (not easy at all) to 5 (very easy). The second part of the study was the remote interview, in which participants were asked to recount their experience using the mobile image capture app and share insights and pain points. While this feedback was qualitative in nature, the full body of data (responses) was quantitatively analyzed to uncover patterns in user feedback.

### Ethical Considerations

The study was approved by the Advarra Institutional Review Board (Pro00074163) and conducted according to the principles of the International Council for Harmonisation of Technical Requirements for Pharmaceuticals for Human Use Guidelines for Good Clinical Practice [22] and the Declaration of Helsinki [23]. All participants provided written informed consent and were compensated with giftcards in the amount of $100 for study participation. The study data contains identifying information which was encrypted. Images were captured and delivered to a secured GXP-compliant system under consistent review of compliance. Event logging on this system ensures the data is tracked and all interactions monitored to ensure safety of study subject data.

## Results

### Mobile App Development

**Figure 1.**
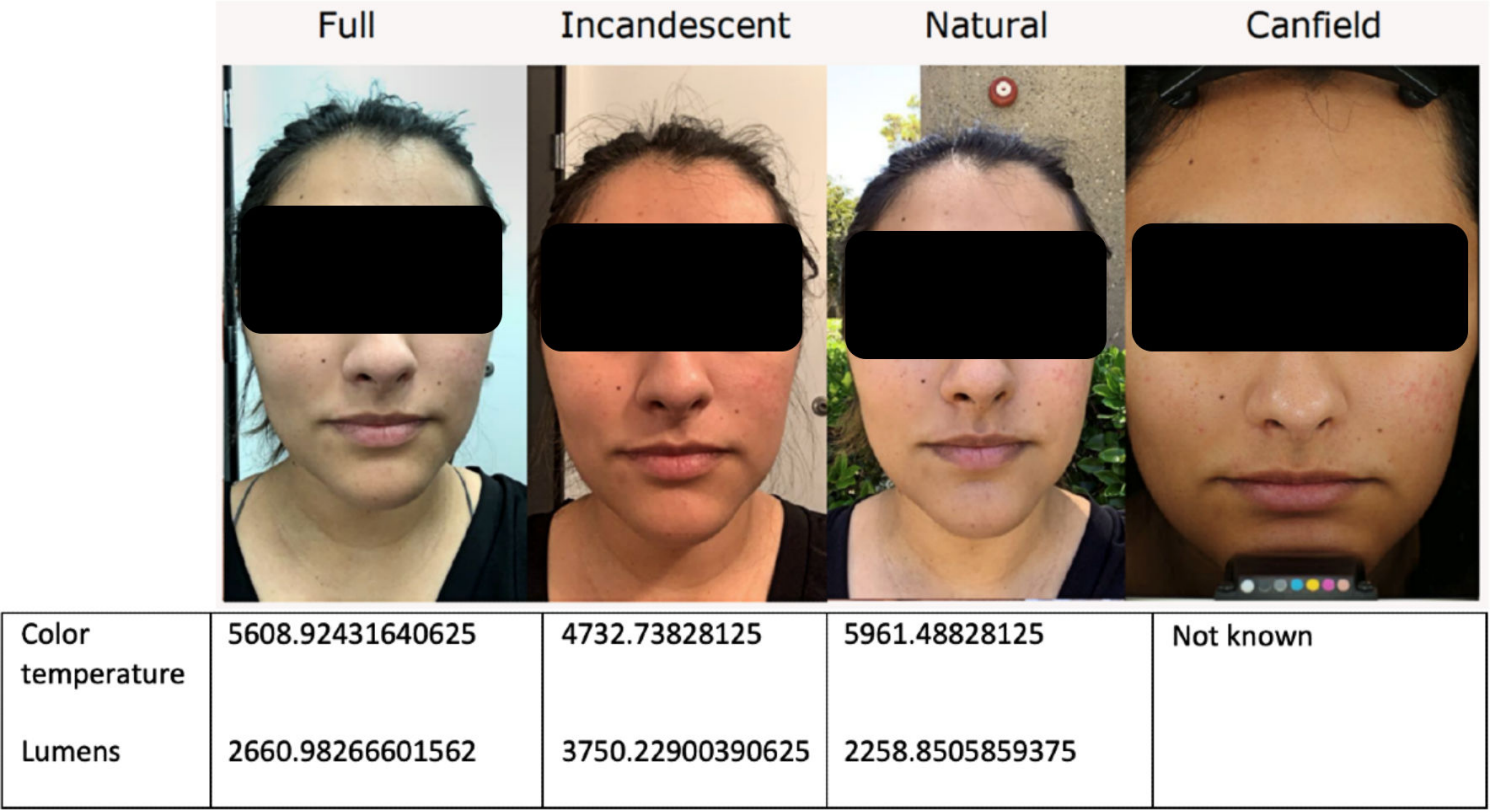
Examples of images captured by a participant (25-year-old Hispanic female, Fitzpatrick skin type IV) using the Allergan Aesthetic mobile image app with an iPhone XR under different lighting conditions (full overhead light, incandescent light, and natural light) compared with a Canfield VISIA-CR system.

Figure 1 shows typical results obtained with the Allergan Aesthetic mobile image app under the different lighting conditions tested, compared with an image from a Canfield system using cross-polarized light. Natural light provided the closest match to that obtained using the in-office Canfield system. However, there was some variability with images taken outdoors depending on the orientation of the participants relative to the sun and the degree of cloudiness.

**Figure 2.**
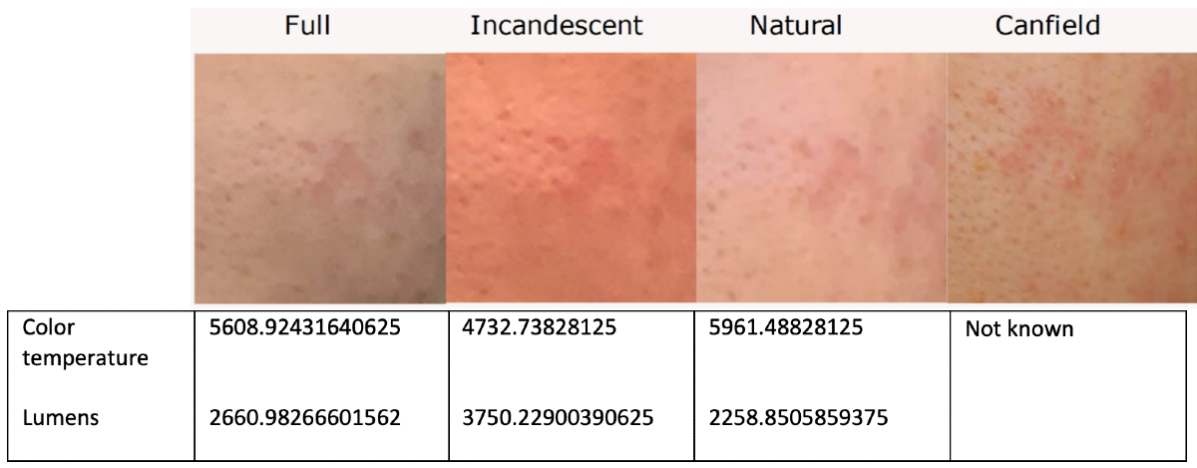
Close-up images of a pimple captured by a mobile device used with the Allergan Aesthetic mobile image app under different lighting conditions (full overhead light, incandescent light, and natural light), showing lower resolution compared with an image captured using a DSLR camera with the Canfield VISIA-CR.

The resolution of the mobile device used had an impact on the quality of images produced under different lighting conditions. As illustrated in Figure 2, a pimple with a white cap is clearly shown with the Canfield system. However, the coloring under incandescent light conditions consistently masked such features that were otherwise clearly identifiable in other modes. Although the redness of the pimple is evident with full light and natural light, the white cap is not visible due to the limited resolution.

**Figure 3.**
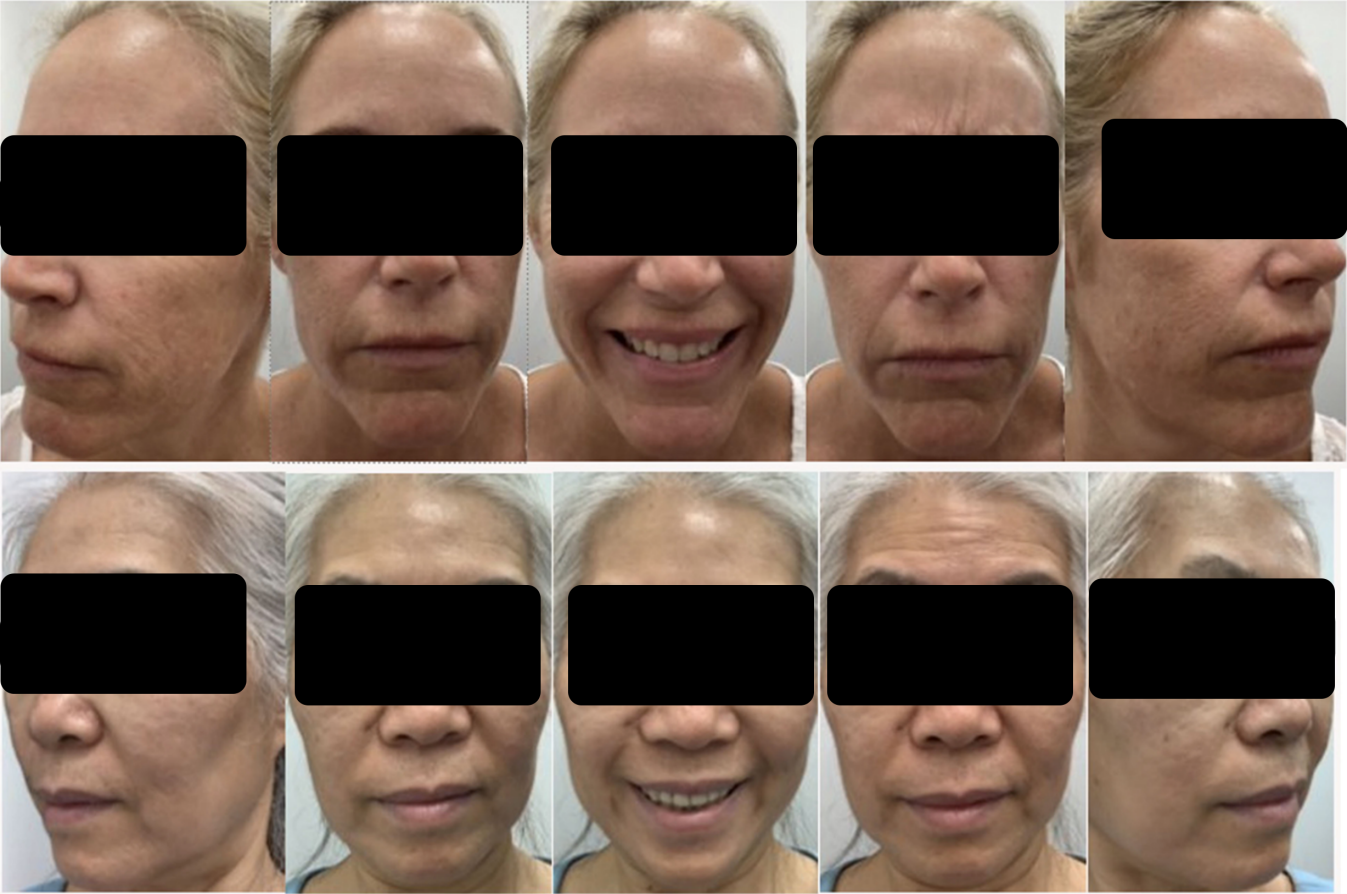
Example image series captured by 2 participants (top row: 48-year-old Caucasian female, Fitzpatrick skin type II; bottom row: 62-year-old Asian female, Fitzpatrick skin type III) with the Allergan Aesthetic mobile image app using an iPhone 12 (top row) and an iPhone XR (bottom row), demonstrating the reproducibility of face position with different angles and expressions. Participants were instructed to follow the positioning and facial-expression prompts provided by the app during auto-capture. The on-site clinician provided additional instruction on how to hold and position the device (ie, hold the phone with 2 hands and place elbows on the table or against the body to stabilize the captures).

The optimal distance from the face to capture images was determined to be 6 inches, as this allowed for optimal resolution and cropping while permitting the participants some latitude to sway from side to side in the image frame. The app was further designed to limit the amount of head rotation and tilt. As illustrated in Figure 3, this results in consistent image capture between participants.

### Image Quality Assessment

A total of 3968 images were assessed using BRISQUE. Overall, BRISQUE scores ranged from −0.24 to 61.83. As illustrated in Table 2, image sets 1 and 2 (captured with the Allergan Aesthetic mobile image app) had lower mean BRISQUE scores of 14.05 and 19.81, respectively, compared with image sets 3 and 4 (captured with Canfield VISIA-CR with a DSLR and the Canfield mobile image capture app, respectively), which had mean scores of 34.47 and 23.43, respectively.

**Table 2.** Comparison of image quality as assessed by BRISQUE^a^ for the Allergan Aesthetic mobile image app and other image capture modalities.

	Image set 1: Allergan Aesthetic mobile image app v1 with iPhone XR	Image set 2: Allergan Aesthetic mobile image app v1 with iPhone 12	Image set 3: Canfield VISIA-CR blue v7.5.4 with DSLR^b^ Canon EOS Rebel T3i	Image set 4: Canfield mobile image capture app with a variety of Android and iOS devices
Image count	560	494	134	2444
Mean	14.05	19.81	34.47	23.43
Minimum	7.00	11.89	18.55	−0.24
Maximum	32.08	30.94	61.83	62.51
Minimum and maximum range	25.08	19.05	43.28	62.75

a BRISQUE: Blind/Referenceless Image Spatial Quality Evaluator.

b DSLR: digital single-lens reflex camera.

### Medical Professional Clinical Analysis

A total of 208 participants were screened for LCL live scale validation; 71 were randomized, out of which 68 (96%) completed the study. Similarly, 208 participants were screened for FHL live scale validation; 73 were randomized, out of which 71 (97%) completed the study. Participants’ characteristics are summarized in Table 3.

The reliability of image assessments of the severity of LCL and FHL using standard digital photography and the participants’ self-capture app is summarized in Table 4. Evaluation of the severity of LCL using independent photo review of standard digital photograph assessments showed that intrarater reliability was almost perfect (overall weighted κ=0.87‐0.88 at rest and 0.89‐0.94 at maximum contraction), and interrater reliability was substantial to almost perfect (ICC=0.73‐0.88 at rest, ICC=0.83‐0.86 at maximum contraction). Interrater reliability between clinician live assessments and independent photo review of standard digital photograph assessments was moderate to almost perfect for both raters (ICC=0.42‐0.89 at rest, ICC=0.49‐0.89 at maximum contraction). For independent photo review of participants’ self-capture photograph assessments, intrarater reliability (overall weighted κ=0.93‐0.94 at rest and 0.96 at maximum contraction) and interrater reliability (ICC=0.89‐0.91 at rest, ICC=0.91‐0.95 at maximum contraction) were almost perfect. Interrater reliability between clinician live assessments and independent photo review of participants’ self-capture photograph assessments was substantial to almost perfect for both raters (ICC=0.75‐0.91 at rest, ICC=0.79‐0.89 at maximum contraction).

Similar results were observed for the assessment of FHL severity. For independent photo review of standard digital photograph assessments, intrarater reliability (overall weighted κ=0.92 at rest and 0.91 at maximum contraction) and interrater reliability (ICC=0.90‐0.91 at rest, ICC=0.86‐0.94 at maximum contraction) were almost perfect. Interrater reliability between clinician live assessments and independent photo review of standard digital photograph assessments was substantial to almost perfect for both raters (ICC=0.77‐0.92 at rest, ICC=0.61‐0.87 at maximum contraction). For independent photo review of participants’ self-capture photograph assessments, intrarater reliability (overall weighted κ=0.94 at rest and 0.92 at maximum contraction) and interrater reliability (ICC=0.91‐0.95 at rest, ICC=0.90‐0.92 at maximum contraction) were almost perfect. Interrater reliability between clinician live assessments and independent photo review of participants’ self-capture photograph assessments was substantial to almost perfect for both raters (ICC=0.77‐0.93 at rest, ICC=0.70‐0.89 at maximum contraction).

**Table 3.** Characteristics of participants undergoing LCL and FHL severity rating (via captured images and in person) in the clinical analysis study.

Characteristic	LCL^b^ (n=68)	FHL^a^ (n=71)
Age (y), median (IQR)	56 (21-89)	52 (20-78)
Female sex, n (%)	52 (77)	48 (68)
White race, n (%)	53 (78)	53 (75)
Fitzpatrick skin type, n (%)		
I/II	36 (53)	29 (41)
III/IV	20 (30)	27 (38)
V/VI	12 (18)	15 (21)

a FHL: forehead lines.

b LCL: lateral canthal lines.

**Table 4.** Evaluation of standard digital photographs and self-capture photographs obtained using the Allergan Aesthetic mobile image app.

Areas of interest	LCL^a^ (n=68)	FHL^b^ (n=71)
Independent photo review of standard digital photographs
Intrarater reliability (overall weighted kappa)
At rest	0.87‐0.88	0.92
At maximum contraction	0.89‐0.94	0.91
Interrater reliability (ICC^c^)
At rest	0.73‐0.88	0.90‐0.91
At maximum contraction	0.83‐0.86	0.86‐0.94
Interrater reliability between clinician live assessments and independent photo review (ICC)
At rest	0.42‐0.89	0.77‐0.92
At maximum contraction	0.49‐0.89	0.61‐0.87
Independent photo review of participant self-captured photographs
Intrarater reliability (overall weighted kappa)
At rest	0.93‐0.94	0.94
At maximum contraction	0.96	0.92
Interrater reliability (ICC)
At rest	0.89‐0.91	0.91‐0.95
At maximum contraction	0.91‐0.95	0.90‐0.92
Interrater reliability between clinician live assessments and independent photo review (ICC)
At rest	0.75‐0.91	0.77‐0.93
At maximum contraction	0.79‐0.89	0.70‐0.89

a LCL: lateral canthal lines.

b FHL: forehead lines.

c ICC: intraclass correlation.

### Usability

In total, 2 rounds of usability research were conducted with a total of 12 participants in the first round and 10 participants in the second round. Although users had some difficulties using the app during the first round of testing, iterative improvements in response to these issues resulted in a much more usable app. In the second round of testing, users rated the app above average in terms of how easy the app experience was to complete (mean rating 3.2 out of 5), how enjoyable the app experience was (3.1), satisfaction with the level of guidance provided (3.2), and likelihood of completing a full session without exiting (4.1). The second round of testing will be used as future inputs for continued refinements to the overall user experience.

## Discussion

### Principal Findings

The use of photography for planning, documentation, and follow-up evaluation of procedures has been a mainstay of aesthetic medicine for decades [2-4]. Clinical practices commonly use specialized equipment and complex setups to maximize image quality and consistency [3,7,8,10,11]; however, in the setting of a clinical trial, the resources and repeated office visits required for this approach can be limiting. The Allergan Aesthetic mobile image app was developed to facilitate clinical trial data collection by making it possible for patients to perform remote self-capture of clinical-grade facial images. In this analysis, the app was found to provide high-quality, reproducible images. Furthermore, participants were satisfied with the app’s ease of use as well as the overall user experience.

The image resolution of a mobile phone is somewhat less than that available with a DSLR, with 7‐12 megapixels for the iPhones used in this study versus 20.2 megapixels for the Canon EOS 6D DSLR, and a pixel size of 1.4 μm versus 6.54 μm, respectively. While this may limit the utility of the app for assessing smaller structures such as pores, pimples, and fine lines, it is adequate for the assessment of larger structures, such as glabellar lines and FHL. When the app was used in an at-home setting, natural light was found to be superior to incandescent light due to the reddish pall conferred by the indoor lighting. However, different lighting conditions may be of benefit depending on the indication for the photograph.

The image quality achieved with a mobile phone using the Allergan Aesthetic mobile image app was on par with professional image capture systems widely used in aesthetic medicine trials, even surpassing that achieved using Canfield VISIA-CR with a DSLR, as assessed by BRISQUE analysis. Furthermore, image quality was very consistent, with a narrow range of image quality scores compared with that seen with Canfield used in combination with both a DSLR and a mobile device. This finding may prove to be more important than overall quality because consistency is key when collecting images in a study for clinical analysis by medical professionals. However, while the use of BRISQUE for quality assessment is directional in nature, we would refrain from using it as an analysis tool in the future, given the weight and superiority of clinical analysis.

The primary evaluation of the Allergan Aesthetic mobile image app is its utility in a clinical setting. Assessment of LCL and FHL by trained raters in images captured using the app was highly consistent both by the same rater and between raters. Importantly, results were also consistent with in-person assessments of LCL and FHL. Although not directly compared, the clinical utility of images captured using the app was similar to that achieved with images captured using standard digital photography. Taken together, these findings indicate that the app is capable of providing clinically relevant results, offering flexibility in the execution of future clinical trials. In addition to providing high-quality, reproducible images, the mobile technology described in this report may help improve diversity in aesthetic clinical studies by allowing enhanced remote prescreening and removing barriers to participation, for example, by minimizing required clinical site visits, which can encumber participation in underserved communities [24]. To our knowledge, the Allergan Aesthetic mobile image app is one of the first apps shown to enable the capture of patient images suitable for use in clinical dermatology research. A recent report from Jin et al [25] describes the development of SkinTracker (RedBlink Inc), a mobile app for integrated collection of patient skin photos, patient-reported outcomes, and biometric data. Results from a pilot study suggest that SkinTracker has the potential to facilitate the collection of patient data in clinical trials of atopic dermatitis and other skin conditions; however, the consistency of ratings obtained via assessment of images captured using SkinTracker versus standard photography or in-person assessment has not yet been examined [25].

The Allergan Aesthetic mobile image app was developed to provide a high level of reproducibility and broad applicability, which requires an information framework designed to support the user’s mental model and deliver actionable information. Mental models are an individual’s representation of reality and aim to foresee how users will interact with a product [26]. Although selfie images appear to be the norm today, mobile image capture (a selfie in its simplest sense) is still a new behavior for users, requiring users to accomplish a certain level of learning while simultaneously performing a series of tasks to ensure standardization. Developing a complex application of this sort is an iterative process. In just 2 iterations of development and testing of the user experience, success was achieved with the second round of testing showing that users had an overall positive experience with the app, returning above-average ratings for all metrics tested. While the latest version of the app appears to be acceptable, there remains an opportunity to further improve the app by providing more guidance to ensure a smoother and more enjoyable experience for all users.

A limitation of this research is that the population enrolled lacked diversity; for example, gender assignment in this study was binary. Future studies should include gender-diverse participants. In addition, the study population was predominantly female (68% to 77%) and White (75% to 78%), with 18% to 21% of patients having Fitzpatrick skin types V/VI. Skin color and anatomical variations according to gender, race, ethnicity, and age can contribute to inaccuracies in image analysis [27-29]. Consequently, it will be critical to further validate the Allergan Aesthetic mobile image app in various populations, including by race, sex, age, and severity of LCL and FHL. However, it is noteworthy that despite this potential limitation, this study was still more diverse than historical aesthetic studies; by comparison, typical reported clinical trial populations are approximately 80% to 94% female, approximately 87% to 98% White, and <5% Fitzpatrick skin types V/VI [30-35]. Another limitation is that we could not control all lighting scenarios, so there was a fair degree of variability in lighting. However, the favorable intra- and interrater agreement indicates that the app is largely able to overcome this problem, which can be considered a positive given the natural variability that will exist in real-world apps.

### Conclusion

The Allergan Aesthetic mobile image app delivers consistent, clinical-grade facial images that can be effectively used for remote assessment of LCL and FHL and yield rater assessments comparable with that of in-person visits. Users had little difficulty using the app, and future iterations will aim to make the experience even smoother and more enjoyable. The Allergan Aesthetic mobile image app has the potential to transform the conduct of clinical trials in aesthetic medicine by reducing the burden on clinic resources, increasing data collection opportunities and quality, and lowering barriers for future study participants.

## Supplementary material

10.2196/64764 Multimedia Appendix 1 User rating questions in usability survey**.**

## Abbreviations

BRISQUE: Blind/Referenceless Image Spatial Quality Evaluator
DSLR: digital single-lens reflex
FHL: forehead lines
FHLSS: forehead lines severity scale
ICC: intraclass correlation coefficient
IEC: International Electrotechnical Commission
ISO: International Organization for Standardization
LCL: lateral canthal lines
LCLSS: lateral canthal lines severity scale
