# Supplementary material for: Capturing Optimal Mobile 2D Facial Images in Remote Aesthetics Medicine Clinical Trials: Technical Considerations for Facial Severity Analysis
Source: JMIR Form Res. 2026 Jan 28;10:e64764. doi: 10.2196/64764 (PMC12851522; doi:10.2196/64764)
Supplement: Multimedia Appendix 1 [file formative-v10-e64764-s001.docx]

**Multimedia Appendix**

**Multimedia Appendix 1: User Rating Questions in Usability Survey**

1. Overall, how easy would you say the app experience was to complete?
2. Overall, how enjoyable was the app experience?
3. Overall, how satisfied were you with the level of guidance provided?
4. Overall, how likely were you to complete a full session without exiting?
